# Age estimation in three distinct east Asian population groups using southern Han Chinese dental reference dataset

**DOI:** 10.1186/s12903-019-0942-y

**Published:** 2019-11-11

**Authors:** Jayakumar Jayaraman, Hai Ming Wong, Graham J. Roberts, Nigel M. King, Hugo F. V. Cardoso, Pavethynath Velusamy, Ronaldo G. Vergara, Keni-ichi Yanagita, Teekayu P. Jorns

**Affiliations:** 1 Department of Developmental Dentistry, University of Texas Health School of Dentistry, Texas, USA; 2 Paediatric Dentistry & Orthodontics, Faculty of Dentistry, The University of Hong Kong, 2/F Prince Philip Dental Hospital, 34 Hospital Road, Hong Kong, Hong Kong; 3 Department of Orthodontics, King’s College London Dental Institute, London, UK; 4 Oral Development and Behavioural Sciences, Faculty of Health and Medical Sciences, University of Western Australia, Nedlands, Australia; 5 Department of Archaeology, Simon Fraser University, Burnaby, BC Canada; 6 Department of Orthodontic Science, Tokyo Medical and Dental University, Tokyo, Japan; 7 Section of Orthodontics, Graduate School of Dentistry, University of the East, Manila, Philippines; 8 Department of Pediatric Dentistry, Kyushu University, Fukuoka, Japan; 9 Orofacial Pain Unit, Department of Oral Biology, Faculty of Dentistry, Khon Kaen University, Khon Kaen, Thailand

**Keywords:** Age estimation, Dental age, Southern Chinese, Philippines, Thailand, Japan

## Abstract

**Background:**

Dental age estimation can assist in the identification of victims following natural disasters and it can also help to solve birth date disputes in individuals involved in criminal activities. A reference dataset (RDS) has been developed from the dental development of 2306 subjects of southern Han Chinese origin and subsequently validated. This study aimed to test the applicability of the southern Han Chinese dental maturation RDS on three distinct East Asian population groups.

**Methods:**

A total of 953 dental panoramic radiographs of subjects aged 2 to 24 years were obtained from Philippines, Thailand and Japan. The staging of dental development was conducted according to Anglo-Canadian classification system. The dental age (DA) was calculated using six methods; one un-weighted average and five weighted average (n-tds, sd-tds, se-tds, 1/sd-tds, 1/se-tds) methods based on the scores of the southern Han Chinese RDS. Statistical significance was set at *p* < 0.05 and the variation between chronological age (CA) and DA was evaluated using paired t-test and Bland & Altman scatter plots.

**Results:**

From six dental age calculations, all methods of DA accurately estimated the age of Japanese and few methods in Filipino subjects (n-tds, 1/sd-tds, 1/se-tds). There was consistent overestimation of age for all the methods for Thai females (*p* < 0.05).

**Conclusions:**

The southern Han Chinese dental reference dataset was shown to be most accurate for Japanese, followed by Thai males and it was particularly ineffective for Filipinos and Thai females.

## Background

East Asia comprises of five regions in Asia including Central Asia, Western Asia, South Asia, Southeast Asia, and East Asia. Philippines, a south east Asian country is one of the most disaster-prone countries in the world. The island nation is located on the Pacific Ocean’s ring of fire causing volcanic eruptions and earthquakes, and the country absorbs the abuse of approximately 25 tropical storms and typhoons each year. In 2013, Super Typhoon Yolanda (Haiyun) ravaged various parts of the country that resulted in the death of approximately 5000 people making it one of the deadliest typhoons in the history [1]. Moreover, latest statistics from the United Nations Children’s Fund (UNICEF) states that in the Philippines, only 90% of children under the age of five had their births registered [2] creating various social problems as a result of uncertainty about the exact age of individuals. Similarly, Thailand has also recorded highest number of natural disasters. Between the year 1980 and 2000, around 64 million people were affected by various forms of natural disasters including earthquake, landslide and flood [3]. In 2004, a powerful earthquake struck the northern Sumatra region triggering a Tsunami that killed more than 4000 people and left another 4000 people missing. Dental records were reported to be highly useful for identification of victims following the Tsunami in Thailand [4]. Japan is comprised of three main islands that are located in the eastern coast of Asia constituting approximately 10% of active volcanoes in the world. More than 1500 earthquakes have been recorded every year making it the country with the largest number of earthquakes in the world [5]. In 2011, approximately 18,000 people lost their lives following the devastating effect of the Tohoku earthquake and subsequent Tsunami effects [6].

Forensic age estimation can assist in the identification of victims following natural disasters and it can also help to solve birth date disputes following criminal activities and provide appropriate age-related sentencing. Population-specific methods for age estimation are often preferred by forensic scientists because the reference sample is the one that closely represent the target individuals [7]. Unfortunately, for a myriad of reasons, population-specific methods are not available for all population groups and the experts has to often rely on methods developed on distinct reference samples [8–10]. Although it is unclear whether the matching between the target individuals and the reference sample is a result of genetic, ontogenetic or environmental influences, the problem of determining the method or methods that accurately estimate age of the target individuals still persists.

Very few dental age estimation methods have been developed in East Asia [11, 12], a wide geographical area with people belonging to different cultures, and with different social practices, and languages. The southern Han Chinese reference dataset [13] is one of the reference standards that is currently available for dental age estimation in this part of world. It is important to understand how this dataset can aid in the estimation of age of other East Asian population groups. Previous studies have shown that dental development differs between the southern Han Chinese, the French-Canadian and the United Kingdom population [14, 15], with methods based on the former two groups providing inaccurate age estimations in the southern Han Chinese sample. Following this inaccuracy, a reference dataset has been developed and validated for the southern Han Chinese [13]. Recently, this dataset was tested on a sample of northern Chinese population and found to accurately estimate the age [16]. It is unclear whether the southern Han Chinese dataset can provide reliable dental age estimates in children of three distinctly located countries in East Asia including Japan, Philippines and Thailand. This study would provide much needed test of the cross-population applicability of dental age estimation methods and can provide significant information to aid experts in making decisions about the methods they should employ in forensic and legal circumstances. Hence, this study aims to test the applicability of southern Han Chinese dental reference dataset on three distinct populations in East Asia namely Filipino, Thai, and Japanese.

## Methods

### Ethics approval and consent to participate

**Fig. 1:**
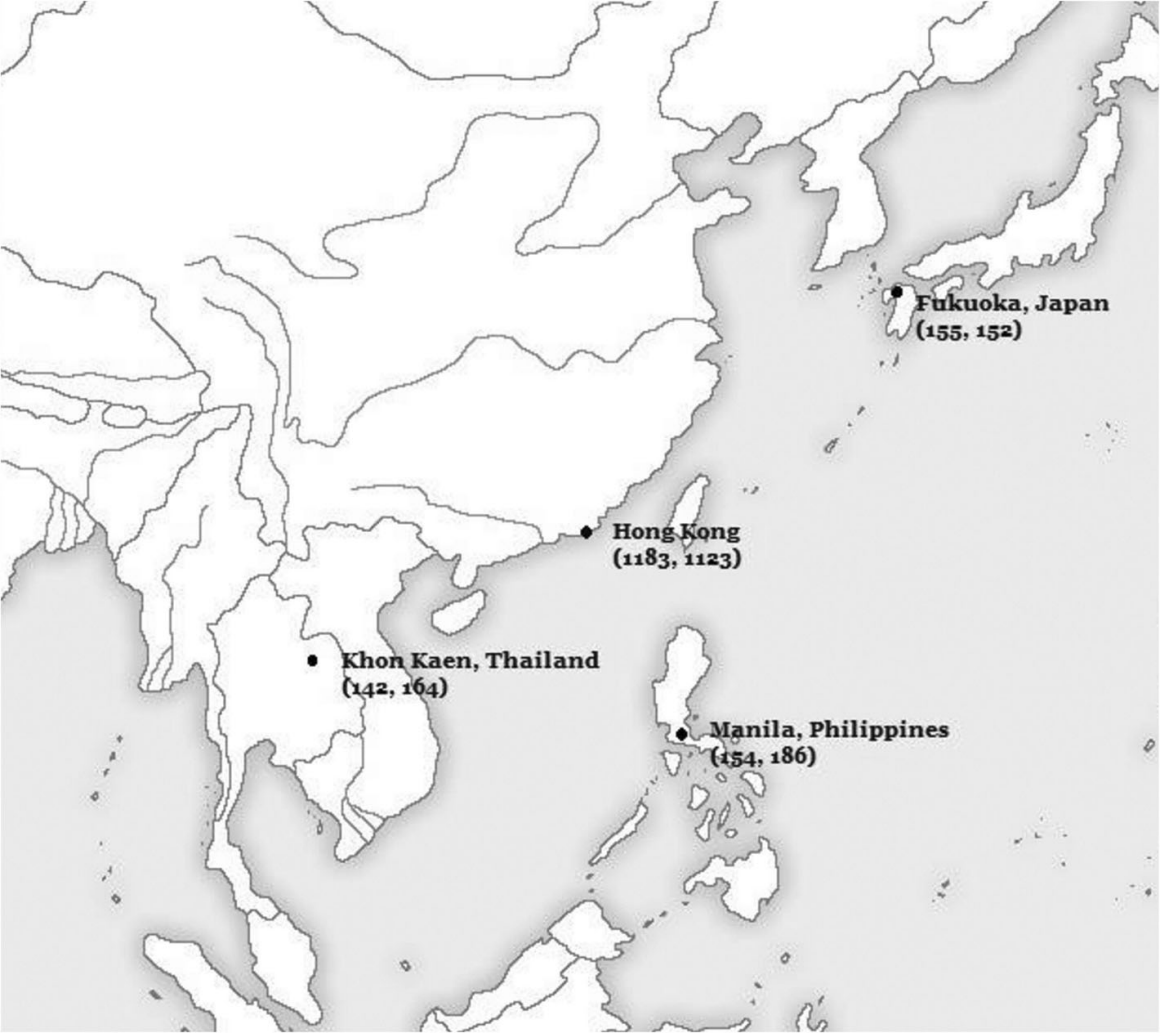
Distribution of samples (males, females) based on their geographical location

Ethical clearance for this study was obtained prior to the start of this study from the University of Hong Kong West Cluster (UW-12-280). A written consent from 18 years or older subjects, and parents/primary caregivers of subjects younger than 18 years old, were obtained from all participants. The southern Chinese dental reference dataset was based on sample of 2306 subjects who were ethnically southern Han Chinese. It comprised of 1123 females and 1183 males aged 2 to 24 years. This dataset had already been evaluated for its accuracy in the same population group [13]. To test the applicability of the southern Han Chinese reference dataset, a total of 953 subjects were obtained from three countries in the East Asian regions; south west (Thailand), south east (Philippines) and north east (Japan). A local ethics committee ruled that no formal ethics approval was required in this particular case. Dental panoramic radiographs of the subjects were randomly selected from the dental teaching hospitals of respective countries. This comprised of 306 subjects from the Faculty of Dentistry, Khon Kaen University, Khon Kaen, Thailand, 340 subjects from the Faculty of Dentistry, University of East, Manila, Philippines and 307 from Faculty of Dental Science, Kyushu University, Fukuoka, Japan (see Table 1)*.* The distribution of samples and their exact geographical locations are illustrated in Fig. 1*.* The subjects included in the study were healthy individuals without any medical conditions that might affect the dental development. The ethnicity of each subject included in the study was verified from their surnames as indicated in the hospital records. In addition, ethnicity was validated by uniqueness of the surnames of parents and grandparents to rule out any inter-racial marriage. Records from each individual were obtained from the respective university teaching hospitals. Individuals in the three samples are considered to belong to an overall middle socioeconomic status segment of the population.

**Table 1:** Age and sex distribution of subjects in the reference data and the validation data from three East Asian populations

Age *(years)*	Reference Dataset^a^				Test Samples			
	Southern Chinese		Philippines		Thailand		Japanese	
	M	F	M	F	M	F	M	F
2.00–2.99	52	53	–	–	–	1	1	2
3.00–3.99	46	50	1	–	1	–	16	10
4.00–4.99	50	56	2	–	1	3	21	12
5.00–5.99	98	99	3	6	3	4	11	14
6.00–6.99	58	52	3	6	9	10	12	10
7.00–7.99	50	55	6	3	9	5	11	11
8.00–8.99	49	50	2	5	9	7	8	13
9.00–9.99	49	50	1	6	7	6	11	11
10.00–10.99	48	50	6	7	7	10	9	10
11.00–11.99	57	44	7	6	5	9	11	5
12.00–12.99	47	49	4	10	10	6	8	7
13.00–13.99	51	54	7	10	3	10	4	12
14.00–14.99	53	49	12	10	13	13	9	5
15.00–15.99	44	43	7	7	10	12	5	6
16.00–16.99	42	45	12	15	9	12	4	10
17.00–17.99	47	56	15	15	9	19	2	5
18.00–18.99	70	36	11	14	7	6	4	1
19.00–19.99	32	33	12	19	5	6	2	3
20.00–20.99	51	43	10	8	7	6	2	–
21.00–21.99	54	37	9	11	5	5	4	1
22.00–22.99	40	38	14	14	2	4	–	1
23.00–23.99	43	37	7	12	5	4	–	2
24.00–24.99	52	44	3	2	6	6	–	1
Total	1183	1123	154	186	142	164	155	152

^a^Data corresponding to the Southern Chinese Reference Dataset^7^

A standard protocol of data acquisition of panoramic radiographs included digital images (Gendex Orthoralix 8500 DDE, Illinois, USA) and scanning the hard copies of the radiographs (Philips Orthoralix SD Ceph, Monza, Italy) at a resolution of minimum 300 dpi (Canon Scan 4400F, Canon Corp, US). All the radiographs had 3% imaging magnification and they were viewed on a 27-in. computer monitor at a magnification of 160% (27 IE, Philips, Philips Corp, US). Details including patient information and ethnicity were hidden from the examiner during the scoring process. A single examiner (JJ) scored all the radiographs. The intra- and inter-examiner reliability scores were tested based on the 8-stage tooth development system described by Demirjian and colleagues [17]. The reliability examination was conducted using Kappa analysis at statistical significance level of *p* < 0.05 [18]. Excel spread sheets were prepared and the details including newly assigned ID number, date of birth, date of radiograph, sex and ethnicity were entered (Microsoft Excel, Microsoft Corp, US). For each subject, sex specific scores were identified and the mean Ages of Attainment (AoA) and the associated standard deviations were obtained from the southern Han Chinese reference dataset. Scores corresponding to stage H of tooth development were excluded from the analysis.

A total of six dental age estimation methods were employed; one simple average method (SAM) and five weighted average method (WAM) calculation based on the parameters associated with tooth development stages (TDS) [19]. The choice of the weighted methods was made under the assumption that this method can better reflect the sequence and pattern of variation in dental development that is used for estimating age [19]. A semiautomatic process of calculating weighted averages was prepared in Excel Worksheet. The five weighted average methods were the average determined by using the number of TDS (n-tds), standard deviation of TDS (sd-tds), standard error of TDS (se-tds), reciprocal of standard deviation of TDS (1/sd-tds) and reciprocal of standard error of TDS (1/se-tds) as the weighting factors. Following this, paired t-tests were calculated using SPSS software (SPSS 16.0, IBM Inc., Chicago, IL) to compare dental age (DA) calculated from six methods with chronological age (CA), separately for males and females. Simple differences between chronological age and dental age (CA-DA) were calculated and are provided to show whether dental age provided an overestimation (negative value) or an underestimation (positive value). Statistical significance for the tests was set at probability values of less than 0.05. Furthermore, Bland & Altman plots in the form of scatterplots of CA-DA (y-axis) by CA (x-axis) were generated to visualize the variation in the difference between the dental age and the chronological age (CA-DA) estimated from the weighted average method (1/sd-tds) in the Japanese, Filipino and Thai subjects using the southern Han Chinese reference dataset.

## Results

### Sample characteristics

The mean age (and standard deviation) for males and females in the southern Han Chinese reference dataset were 11.57 (±5.51) years and 11.88 (±5.90) years respectively. In this study, the mean age (and standard deviation) for males and females were 14.53 (±4.57) and 14.56 (±4.46) years for the Filipino subjects, 12.89 (±4.58) and 13.47 (±4.66) years for the Thai subjects, and 9.06 (±4.59) and 9.61 (±4.34) years for the Japanese subjects respectively (Tables 2, 3 and 4).

**Table 2:** Minimum (Min.), maximum (Max.) and mean (DA-CA) difference between the chronological (CA) and dental age (DA) in Filipino males and females using the southern Han Chinese reference dataset and the six different dental age (DA) calculation methods

Age	Males					Females				
	Mean Age	SD Age	Mean CA-DA^	SD CA-DA	t-test	Mean Age	SD Age	Mean CA-DA^	SD CA-DA	t-test
Chronological Age (CA)	14.53	4.57	–	–	–	14.56	4.46	–	–	–
DA - No Weighting [nil]	14.76	4.52	−0.23	1.18	0.045*	14.74	4.64	−0.18	1.11	0.050
DA - Weighted Average [n-tds]	14.72	4.55	−0.19	1.22	0.102	14.74	4.66	−0.18	1.13	0.053
DA - Weighted Average [sd-tds]	14.78	4.51	−0.25	1.19	0.027*	14.78	4.63	−0.23	1.11	0.016*
DA - Weighted Average [se-tds]	14.80	4.50	−0.27	1.19	0.017*	14.78	4.62	−0.23	1.10	0.016*
DA - Weighted Average [1/sd-tds]	14.72	4.53	−0.19	1.18	0.083	14.69	4.65	−0.13	1.11	0.145
DA - Weighted Average [1/se-tds]	14.70	4.54	−0.17	1.19	0.124	14.70	4.66	−0.14	1.12	0.142

DA-Dental Age, *statistically significant value for CA-DA at *p* < 0.05,^negative value indicates overestimation and positive value indicates underestimation of age

**Table 3:** Minimum (Min.), maximum (Max.) and mean (DA-CA) difference between the chronological (CA) and dental age (DA) in Thai males and females using the southern Han Chinese reference dataset and the six different dental age (DA) calculation methods

Age	Males					Females				
	Mean Age	SD Age	Mean CA-DA^	SD CA-DA	t-test	Mean Age	SD Age	Mean CA-DA^	SD CA-DA	t-test
Chronological Age (CA)	12.89	4.58	–	–	–	13.47	4.66	–	–	–
DA - No Weighting [nil]	13.00	4.36	−0.11	0.97	0.245	13.78	4.48	−0.31	1.26	0.003*
DA - Weighted Average [n-tds]	12.97	4.39	−0.08	0.94	0.372	13.78	4.50	−0.31	1.28	0.004*
DA - Weighted Average [sd-tds]	13.03	4.36	−0.14	0.97	0.117	13.82	4.47	−0.35	1.26	0.001*
DA - Weighted Average [se-tds]	13.05	4.35	−0.16	1.00	0.094	13.82	4.46	−0.35	1.25	0.001*
DA - Weighted Average [1/sd-tds]	12.96	4.37	−0.07	0.97	0.443	13.74	4.49	−0.27	1.26	0.012*
DA - Weighted Average [1/se-tds]	12.95	4.38	−0.06	0.95	0.536	13.74	4.50	−0.27	1.27	0.011*

DA-Dental Age, *statistically significant value for CA-DA at *p* < 0.05,^negative value indicates overestimation and positive value indicates underestimation of age

**Table 4:** Minimum (Min.), maximum (Max.) and mean (DA-CA) difference between the chronological (CA) and dental age (DA) in Japanese males and females using the southern Han Chinese reference dataset and the six different dental age (DA) calculation methods

Age	Males					Females				
	Mean Age	SD Age	Mean CA-DA^	SD CA-DA	t-test	Mean Age	SD Age	Mean CA-DA^	SD CA-DA	t-test
Chronological Age (CA)	9.06	4.59	–	–	–	9.61	4.34	–	–	–
DA - No Weighting [nil]	9.00	4.73	0.06	0.85	0.397	9.56	4.44	0.05	0.85	0.542
DA - Weighted Average [n-tds]	9.01	4.72	0.05	0.88	0.445	9.54	4.43	0.07	0.86	0.373
DA - Weighted Average [sd-tds]	9.03	4.72	0.03	0.86	0.656	9.61	4.42	0.00	0.85	0.972
DA - Weighted Average [se-tds]	9.03	4.73	0.03	0.86	0.624	9.61	4.43	−0.01	0.86	0.926
DA - Weighted Average [1/sd-tds]	8.97	4.73	0.09	0.85	0.203	9.52	4.46	0.09	0.85	0.236
DA - Weighted Average [1/se-tds]	8.98	4.73	0.08	0.85	0.228	9.52	4.45	0.09	0.85	0.208

DA-Dental Age, *statistically significant value for CA-DA at *p* < 0.05, ^negative value indicates overestimation and positive value indicates underestimation of age

### Examiner reliability scores

Fifty radiographs were randomly chosen to test the reliability of the examiner (JJ). For inter-examiner reliability, the radiographs were scored by the main examiner (JJ) and the second examiner (HMW). For intra-examiner reliability, the same radiographs were scored by the main examiner (JJ) at two-time intervals, at the beginning of the study and after a period of 3 weeks. The Kappa scores were 0.81 and 0.85 for intra- and inter-examiner reliability and these scores correspond to “almost perfect” observations [18].

### Filipino subjects

Comparing differences between CA-DA, results show that the southern Chinese Han reference dataset overestimated the age of all Filipino subjects. In males, all the dental age calculations over-estimated the age with a minimum difference of − 0.17 years for 1/se-tds method to a maximum difference of − 0.27 years for se-tds method. A similar overestimation was observed in females with a minimum difference of − 0.13 years for 1/sd-tds and 1/se-tds methods and maximum difference of − 0.23 years for sd-tds and se-tds methods. Paired t-test showed significant differences in the estimated dental ages using the se-tds and sd-tds methods in both sexes and the dental age estimated without any weighting factor in males (*p* < 0.05, see Table 3).

### Thai subjects

The method also overestimated chronological age in Thai males and females. The CA-DA difference ranged from − 0.06 years (1/se-tds) to − 0.16 years (se-tds) in males and − 0.27 years (1/sd-tds and 1/se-tds) to − 0.35 years (sd-tds and se-tds) in females respectively. Statistically significant difference was observed between the CA and the DA estimated from the six calculation methods (Table 4). This trend was observed only in females (*p* < 0.05).

### Japanese subjects

The age of Japanese subjects was also overestimated from the method, showing minimal CA-DA differences. CA-DA differences were all statistically insignificant for both sexes (*p* > 0.05), regardless of the method used to estimate dental age and indicating that the southern Chinese RDS reference dataset best matched with the Japanese sample (Table 4), compared to the other population groups. This ranged from 0.03 years for sd-tds and se-tds methods to 0.09 years for 1/sd-tds method in males. Similarly, for females, the difference ranged from − 0.01 years for se-tds method to 0.09 years for 1/se-tds method.

### Bland & Altman scatter plots

**Fig. 2:**
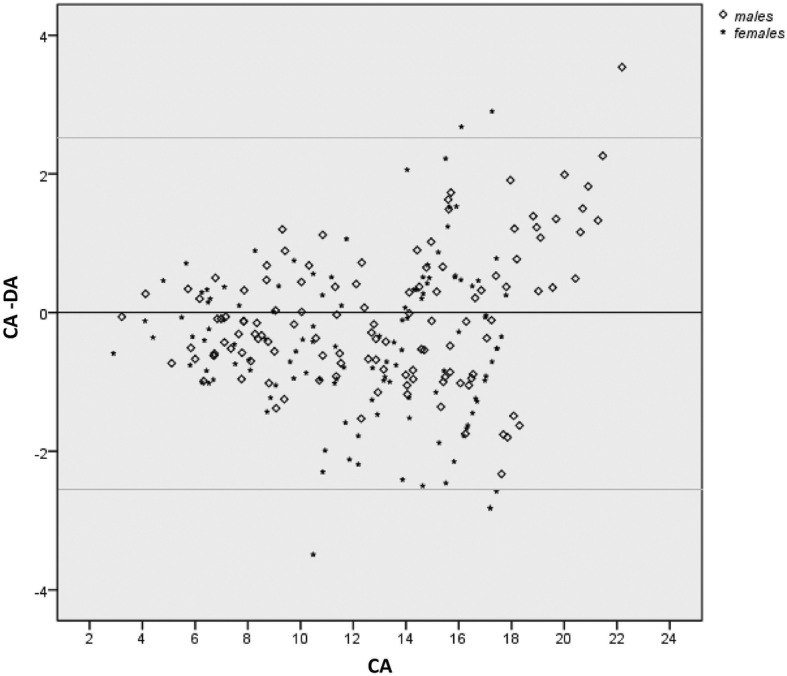
Variations (in years) between the Chronological Age (CA) and estimated Dental Age (1/sd-tds) in the Thai subjects using the southern Han Chinese reference dataset

**Fig. 3:**
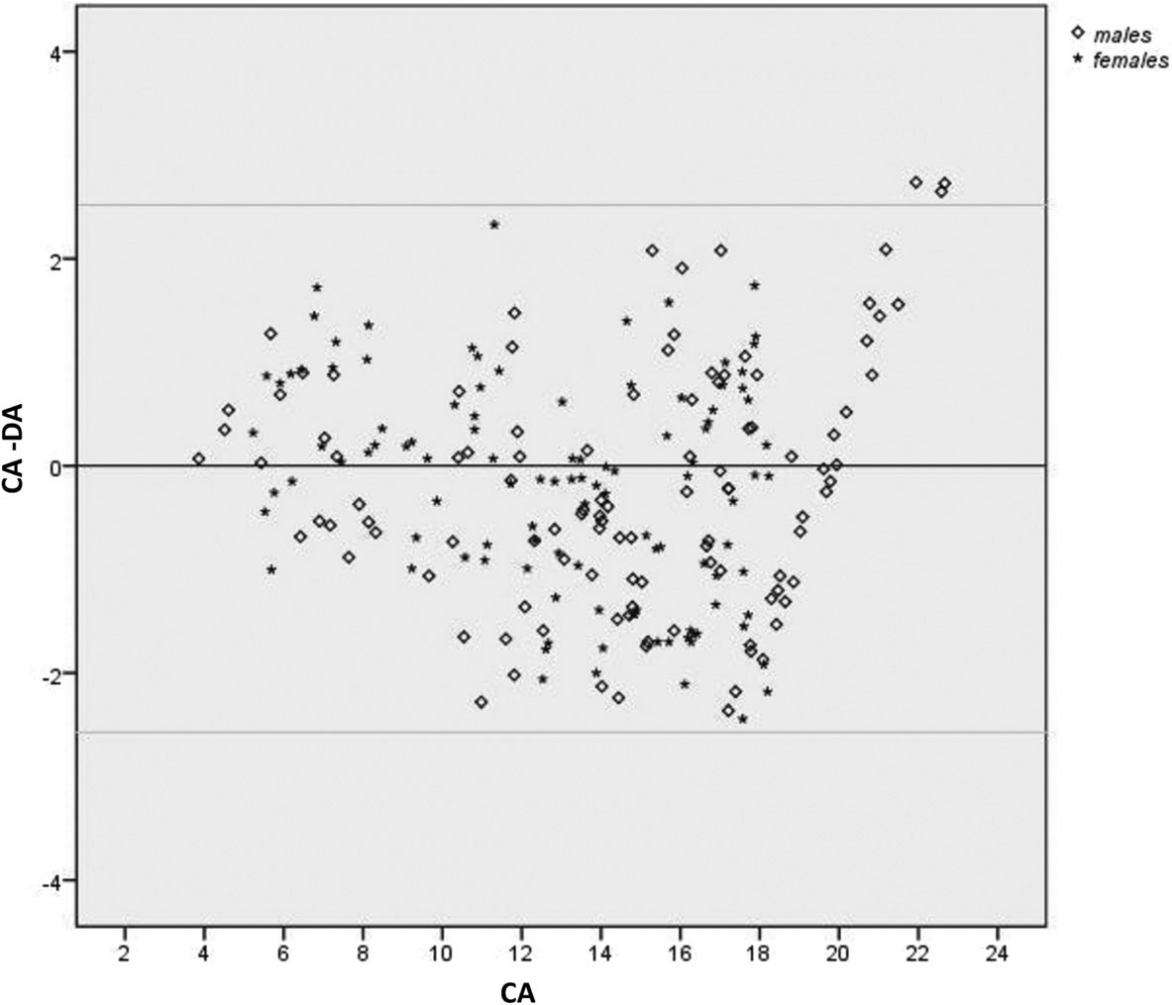
Variations (in years) between the Chronological Age (CA) and estimated Dental Age (1/sd-tds) in the Filipino subjects using the southern Han Chinese reference dataset

**Fig. 4:**
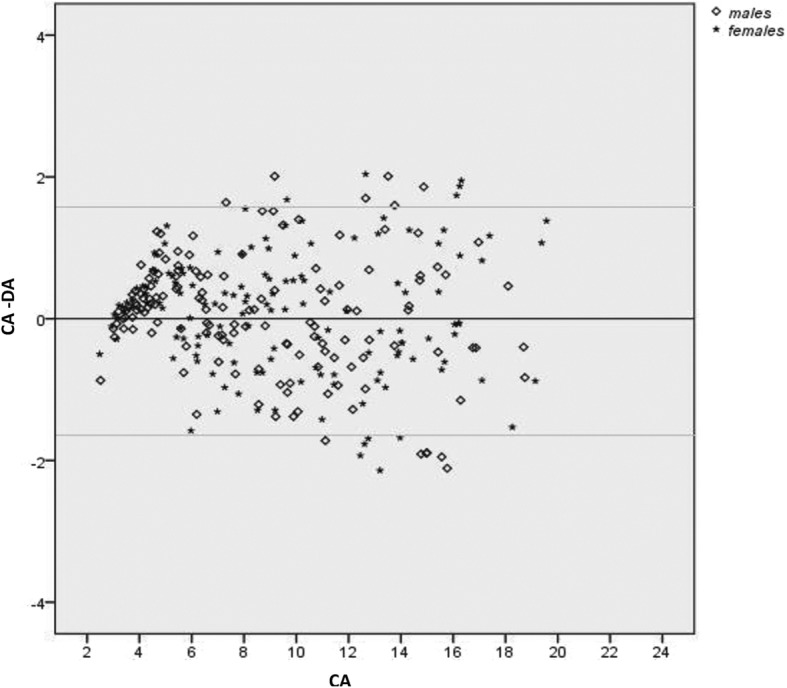
Variations (in years) between the Chronological Age (CA) and estimated Dental Age (1/sd-tds) in the Japanese subjects using the southern Han Chinese reference dataset

Figures 2, 3 and 4 show the variation in the difference between CA and DA (CA-DA) in the y-axis and chronological age (CA) in the x-axis for all three test samples. The 1/sd-tds calculation was chosen to represent the differences since this provided the most accurate age estimate all groups (Tables 2, 3 and 4). The upper and lower limits were marked to represent +/− 2SD of the observed differences between the CA and DA. Maximum variation in age was observed in Thais, followed by Filipinos and Japanese. Figures 2, 3 and 4 illustrate about an equal distribution of individuals above and below zero, with increase in variation with age.

## Discussion

Results from this study show the dental age estimation method based on southern Han Chinese dataset had a diverse performance across the different samples tested. The method consistently overestimated the age of Filipino male and females, whereas on the Japanese sample, the estimated age did not differ significantly from the chronological age, in both sexes. In the Thai, whilst the age of males were accurately estimated, overestimation of age was observed in the females. Whether this indicates a close similarity in dental maturation between the Japanese and the southern Chinese populations due to shared population history or similar growth environments is not known [20]. Conversely, results with the Filipino sample seem to show that shared population history may be actually irrelevant. These results suggest that the expectation of a shared population history does not warrant direct applicability of the method across populations that may be perceived as “similar”. In contrast, it may also be difficult to justify the application of population-specific methods across “similar” populations. For example, in Japanese subjects, no statistically significant difference was observed between the CA and DA estimated from the southern Han Chinese data showing that the reference data is reliable. In contrast, significant difference was observed between CA and DA in Filipino subjects.

Results from this study may be partially explained by different age distributions among the test samples. For example, it has been reported that the number of teeth included in the calculation contributes to the accuracy of the estimated age [21]. This finding was consistent with the current study where the most accurate estimations were observed in younger children, particularly in the Japanese sample. It is to be noted that the samples in Japanese population was skewed towards the younger ages. This was the case with higher number of samples in older age range in the Filipino population. This uneven age distributions among the samples resulted from limitations to access to radiographs from the university-based teaching hospitals in Japan, Philippines and Thailand. This can be considered as a limitation and the results of this study can be further confirmed with equal and possibly, higher number of samples allocated to each age range in the respective populations.

To our knowledge, this is the first dental age estimation study carried out in a Filipino sample although other studies has been conducted in Japanese and Thai. A recent study in Thai children and adolescents employed quadratic regression to develop new prediction models for dental age estimation. The mean difference between CA and DA was − 0.04 years for males and 0.02 years for females [22]. In the current study, the most accurate method was using the reciprocal of standard error of the tooth developmental stages (1/se-tds) which showed the overall mean difference at − 0.06 years for males and − 0.35 years for females, suggesting that the method was not as accurate as that of Duangto and co-workers [22]. Similarly, regression analysis was used to develop prediction models for Japanese subjects aged 1 to 23 years by Ramanan and co-workers [12]. These authors utilized a Belgian reference model and found that the adapted scores produced a difference between CA and DA of − 0.06 and 0.08 years in males and females, respectively [23]. When a Japanese reference model was used, the difference between CA and DA was not significantly reduced (− 0.09 and − 0.06 years for males and females, respectively) [12].

Although a number of studies have documented differences in dental development between different populations [24–26], including differences between UK and French-Canadian datasets with the southern Han Chinese population [14, 15], these have been refuted by some authors who found that the differences in the French-Canadian dental maturity score within certain populations is not because of differences in the timing of tooth formation stages at a population level [27]. The same authors in another study found no significant difference in the estimated mean age between British Caucasians and Bangladeshi populations [28]. Similar results were obtained by Ramanan et al. [12] when testing a Belgian and a Japanese reference model in a Japanese sample. Amidst some of the contradictory evidences in the literature, variation in dental development within East Asians populations is not clearly understood. A recent study based on a large sample of Chinese and Caucasian populations found that the Chinese were dentally advanced compared to Caucasians [29]. It is still unclear whether population differences in dental maturity amongst global populations are due to genetic, environmental or methodological factors. Although this study sought to test the applicability of a southern Han Chinese dataset for age estimation in three other East Asian populations, a better understanding of variation in dental maturation patterns would require a finer level of analysis and consideration of other factors, such as socioeconomic status [30], other potential sources of variation in tooth formation timing, sample composition and other methodological issues, as well as a more diverse set of test samples.

## Conclusions

The southern Chinese reference dataset for the estimation of dental age showed a varied performance when tested on a sample of Thai, Filipino and Japanese individuals. Among the criteria used for age estimation, the reference dataset was shown to be the most accurate for Japanese, followed by Thai males and it was particularly ineffective for Thai females and Filipinos. Although it is unclear whether these differences are due to genetic, environmental or even methodological factors, population variation in dental development needs further exploration. Based on the outcomes of this study, the southern Chinese reference dataset can be recommended for use on Japanese subjects, but with reservations on the Thai and Filipinos.

## Data Availability

The datasets used and/or analysed during the current study are available from the corresponding author on reasonable request.

## Abbreviations

1/sd-tds: Reciprocal of standard deviation of TDS
1/se-tds: Reciprocal of standard error of TDS
AoA: Ages of Attainment
CA: Chronological age
DA: Dental age
n-tds: Average determined by using the number of TDS
RDS: Reference dataset
SAM: Simple average method
TDS: Tooth development stages
UNICEF: United Nations Children’s Fund
WAM: Weighted average method
